# Intraoperative hyperspectral imaging (HSI) as a new diagnostic tool for the detection of cartilage degeneration

**DOI:** 10.1038/s41598-021-04642-5

**Published:** 2022-01-12

**Authors:** Max Kistler, Hannes Köhler, Jan Theopold, Ines Gockel, Andreas Roth, Pierre Hepp, Georg Osterhoff

**Affiliations:** 1 Department of Orthopaedics, Trauma and Plastic Surgery, University Hospital Leipzig, 04103 Leipzig, Germany; 2 Innovation Center Computer Assisted Surgery (ICCAS), University of Leipzig, Leipzig, Germany; 3 Department of Visceral, Transplant, Thoracic and Vascular Surgery, Leipzig University Hospital, Leipzig, Germany

**Keywords:** Medical research, Translational research

## Abstract

To investigate, whether hyperspectral imaging (HSI) is able to reliably differentiate between healthy and damaged cartilage tissue. A prospective diagnostic study was performed including 21 patients undergoing open knee surgery. HSI data were acquired during surgery, and the joint surface’s cartilage was assessed according to the ICRS cartilage injury score. The HSI system records light spectra from 500 to 1000 nm and generates several parameters including tissue water index (TWI) and the absorbance at 960 nm and 540 nm. Receiver operating characteristic curves were calculated to assess test parameters for threshold values of HSI. Areas with a cartilage defect ICRS grade ≥ 3 showed a significantly lower TWI (*p* = 0.026) and higher values for 540 nm (*p* < 0.001). No difference was seen for 960 nm (*p* = 0.244). For a threshold of 540 nm > 0.74, a cartilage defect ICRS grade ≥ 3 could be detected with a sensitivity of 0.81 and a specificity of 0.81. TWI was not suitable for cartilage defect detection. HSI can provide reliable parameters to differentiate healthy and damaged cartilage. Our data clearly suggest that the difference in absorbance at 540 nm would be the best parameter to achieve accurate identification of damaged cartilage.

## Introduction

Cartilage degeneration as seen in osteoarthritis is a common disease, causing pain and disability. It frequently results in a need for prolonged non-operative therapy, ultimately surgical joint replacement, causing a relevant socio-economic burden to the individual and the society^1^.

Imaging modalities currently used to detect cartilage or joint degeneration are radiography and magnetic resonance imaging (MRI). Radiography can only use surrogate parameters for cartilage degeneration, as the joint space width^2^. Those morphological changes occur at later stages of cartilage degeneration only^2,3^. While radiography is still the most common and least expensive technique, MRI is able to visualize the cartilage directly and to show biochemical changes, like an increase of water content of the cartilage tissue in the early stages of degeneration^2–4^. Still, it remains difficult to precisely image the thin articular cartilage and to detect small fibrillations^5^ and initial degenerative changes^6^.

Another method to examine cartilage is arthroscopy. This invasive procedure is necessary, because sometimes, the above-mentioned imaging modalities alone are not sufficient. There are cases, in which changes in cartilage are seen through direct visualization using arthroscopy. However, MRI and the findings of arthroscopy do not always correlate well^7–9^. In such cases, the examiner currently has visual scores at his disposal to objectify those lesions only, e.g. the International Cartilage Repair Society (ICRS)-Cartilage Injury Classification^10^. Because of this and differences in experience, there is a large inter-observer variance in arthroscopy as well as in MRI^6,8^. Initial changes are also difficult to detect by arthroscopy^6^, and sometimes there are already degenerative changes in visually healthy cartilage^11^.

Hyperspectral Imaging (HSI) is a relatively new technique that can show biochemical and morphological information and changes in tissue, even if they are not visible by the surgeon’s judgment. The combination of spectroscopy and imaging in one process allows this^12^. The advantage of the in vivo spectral imaging is that functional and structural pathologic changes of the tissues can be detected at the same time and be used direct grading of tissue damage without the need of a biopsy^13^. Therefore, it is a valuable tool to detect the changes in morphology mentioned above, which occur in degenerated cartilage. It has already been used successfully to detect such changes e.g. in a carcinoma^14–16^ or in ischemic tissue^17–19^, and it has been applied to differentiate tissues based on their spectral properties^20,21^.

The current study aims to investigate whether the HSI technique is able to reliably differentiate between healthy and damaged cartilage tissue, and therefore to detect areas of cartilage degeneration.

## Patients and methods

### Patients

The protocol of this prospective diagnostic study was approved by the ethics committee of the Medical Faculty of the University of Leipzig (reference 393/16-ek). All methods were performed in accordance with the relevant guidelines and regulations. Consecutive patients aged 18 and older, who underwent total or hemiarthroplasty for degenerative osteoarthritis of the knee between March and December 2019 and gave informed consent, were included. In total, 21 patients (19 female) with a median age of 63 years (43 to 84) were included.

### Data acquisition

During surgery and before resection of the joint surfaces, images were taken using the Hyperspectral Imaging camera (TIVITA Tissue, Diaspective Vision GmbH, Germany). The system uses a built-in halogen light source to record reflectance spectra from 500 to 1000 nm in 6.4 s. The field of view (FOV) for all parameters was 21 × 30 cm^2^ and the spatial resolution was 0.56 mm at 630 nm (evaluated with the 1951 USAF resolution test chart at 50 cm object distance). A more detailed description of the used HSI camera system has previously been published by Holmer et al.^22^.

**Figure 1:**
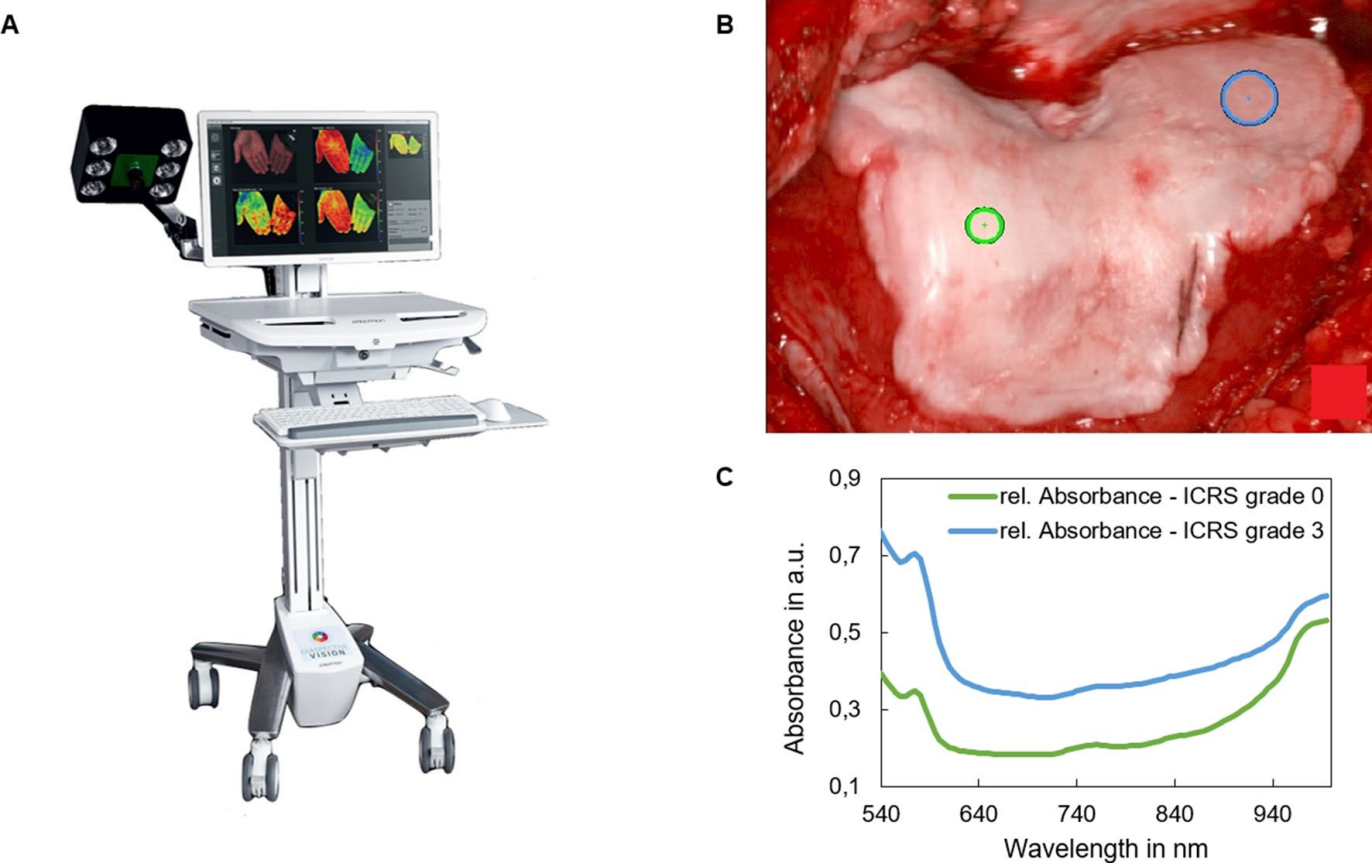
Hyperspectral Imaging-acquisition. (**A**) HSI camera (TIVITA Tissue, image reproduced] from Diaspective Vision GmbH, Germany, all rights reserved). (**B**) RGB image with markers (small green marker: ICRS grade 0, large blue marker: ICRS grade 3). (**C**) Spectral data for cartilage marked ICRS grade 0 and ICRS grade 3. The spectra are calculated as follows: IR = (I0 − Idark)/(Iwhite − Idark), where Idark is the dark pattern without illumination, I0 is the acquired intensity, and Iwhite is the intensity measured on a white reference. The absorbance A was calculated by A = − log10 (IR).

An experienced fellowship-trained surgeon assessed the joint surface and defined areas with a cartilage defect of ≥ 3 according to the International Cartilage Repair Society (ICRS) Cartilage Injury Classification^10^, as well as areas with a cartilage defect of ICRS < 2, serving as healthy control. These areas were manually labelled on the plain red–green–blue RGB image of the joint surface to allow for comparison during analysis of the HSI data later (Fig. 1).

Using the HSI data, tissue water index (TWI), as well as relative absorbance at 960 nm and at 540 nm were chosen from the near-infrared and visible spectrum as candidate parameters. A wavelength of 960 nm was chosen as in the range of 500 nm to 1000 nm, as the absorption coefficient of water shows its maximum at 960 nm. Our selection of parameters was based on previous studies with animal cadaver cartilage specimens^23–26^ and parameters previously described by Holmer et al.^22^ In addition, threshold values for the classification of cartilage defects were identified based on the spectra (Fig. 1C).

TIVITA Suite automatically generates false colour images for physiological tissue parameters, calculating indices, e.g. for oxygenation and perfusion, showing the values from 0 to 100 color-coded. The TWI is calculated for the water content with:

$$ {\text{TWI}}\;{ = }\;\frac{{\frac{{mean \left( {955 - 980 nm} \right)}}{{mean \left( {880 - 900 nm} \right)}} - s1}}{s2 - s1}, $$

s1, and s2 being parameters to scale the values between 0 and 100^22^.

### Statistical analysis

All calculations were performed with TIVITA Suite and SPSS 25.0. Continuous data are presented as mean and standard deviation (SD), categorical data in frequencies and percentages. After testing for normal distribution using the Shapiro–Wilk test, either a paired Student’s-t test or paired non-parametric tests were used to detect differences in means between healthy and damaged cartilage. The level of significance was defined as *p* < 0.05.

Receiver operating characteristic (ROC) tables were computed to examine the sensitivity and specificity of threshold values of the HSI parameters that showed significant differences in means and would correctly indicate a cartilage defect. A priori, we decided that a threshold of > 80% sensitivity and specificity represented a clinically acceptable margin of error, as these are in range of a decile of the values achieved by magnetic resonance imaging (sensitivity 0.72–a0.87, specificity 0.86–a0.89)^27^.

### Ethics approval

All methods were performed in accordance with the relevant guidelines and regulations. The protocol of this prospective diagnostic study was approved by the ethics committee of the Medical Faculty of the University of Leipzig (reference 393/16-ek).

### Consent to participate and publication

All participants gave informed consent to the use of their data and images for analysis and publication.

## Results

### ICRS grading

All specimens contained areas of healthy (ICRS grade < 2) and damaged cartilage (ICRS grade ≥ 3). Two of 21 cartilage defects (9.5%) were ICRS grade 4, all others (90.5%) were grade 3.

### Hyperspectral Imaging

**Figure 2:**
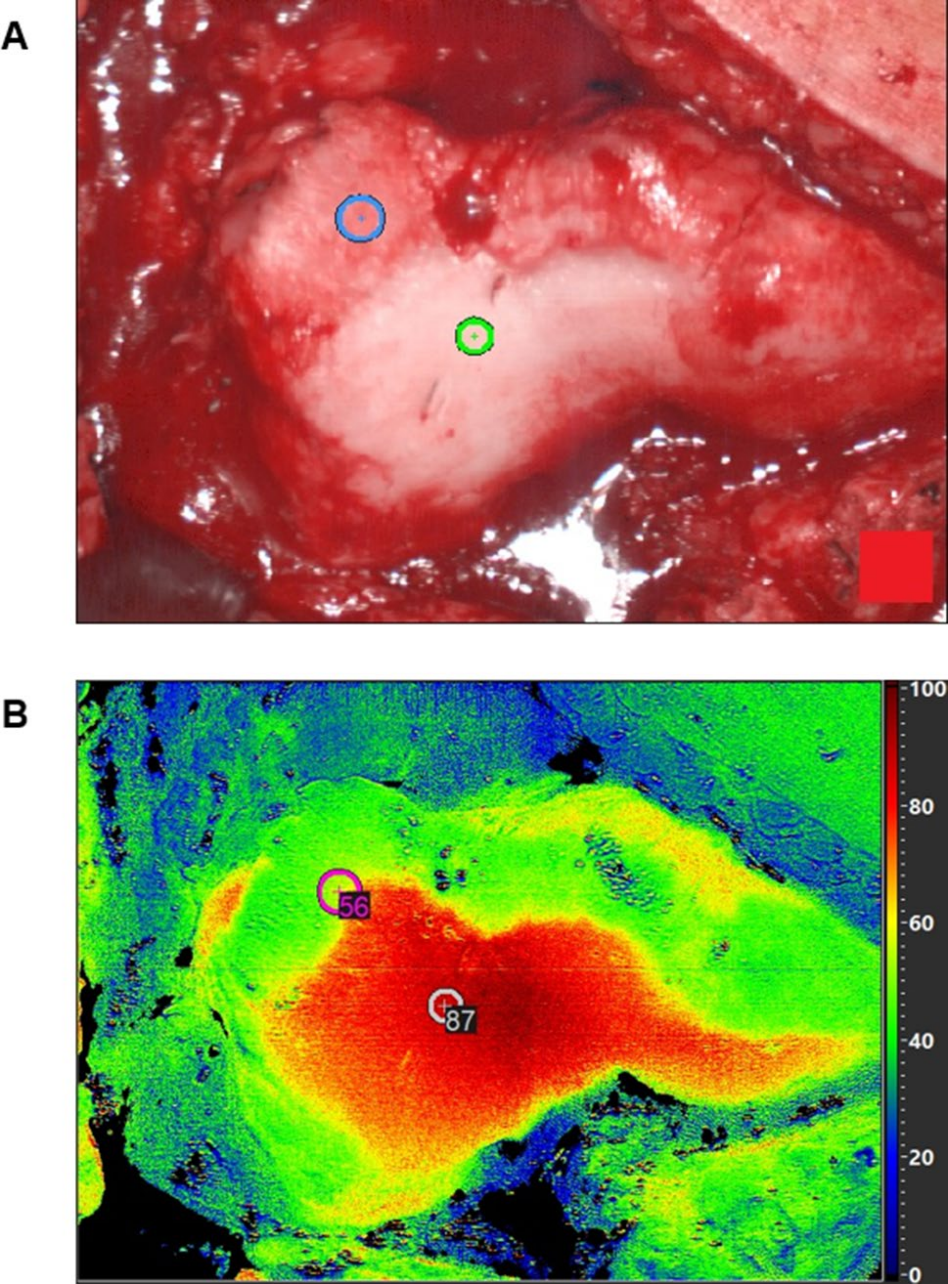
Example of a false color images for the TWI. (**A**) RGB image of the femoral trochlea joint surface. (**B**) Corresponding TWI image with ICRS grade 0 and ICRS grade 3 lesions marked.

Areas with a cartilage defect ICRS grade ≥ 3 showed a significantly lower TWI (*p* = 0.026, Table 1, Fig. 2), and higher values for 540 nm (*p* < 0.001). No difference was seen for 960 nm (*p* = 0.244).

**Table 1:** Relative absorbance and TWI of healthy and damaged cartilage.

	Cartilage		*p*
	Healthy	Damaged	
**TWI**	75 ± 15	67 ± 12	0.026
**540 nm**	0.59 ± 0.27	1.18 ± 0.48	< 0.001
**960 nm**	0.47 ± 0.10	0.49 ± 0.07	0.244

### ROC analysis

**Figure 3:**
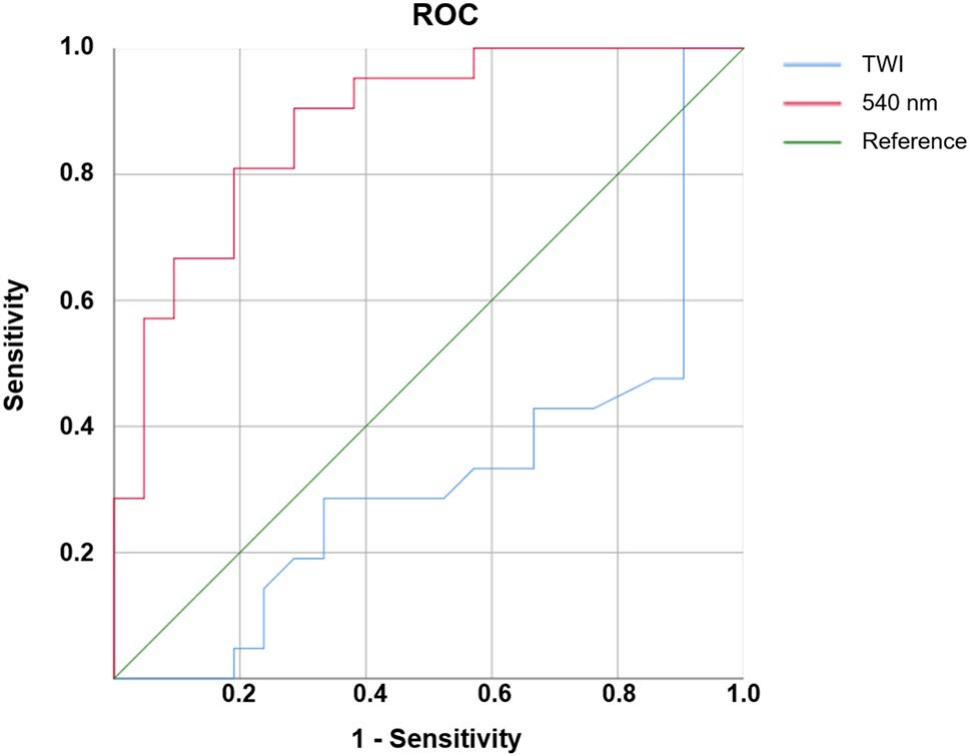
ROC analysis.

As only TWI and 540 nm revealed significant differences in means between healthy and damaged cartilage, ROC tables were computed for these two parameters only. With an area under the curve (AUC) of 0.322, TWI did not prove a relevant parameter for differentiating between healthy and damaged cartilage (Fig. 3).

For 540 nm, however, the logistic regression model showed a good compatibility with an AUC of 0.878.

At a threshold of 540 nm > 0.74, a cartilage defect ICRS grade ≥ 3 could be detected with a sensitivity of 0.81 and a specificity of 0.81.

## Discussion

This study aimed to investigate, whether HSI is able to detect areas of cartilage degeneration reliably by differentiating between healthy and damaged cartilage tissue.

In this prospective diagnostic study, the relative absorbance at 540 nm exerted the potential to differentiate between healthy or damaged cartilage with a sensitivity of 0.81 and a specificity of 0.81. This resembles a diagnostic accuracy close to that of magnetic resonance imaging. While the sensitivity of HSI is comparable to the sensitivity of MRI in detecting cartilage damage reported in the literature (0.72–a0.87), the specificity of HSI is lower than what is reported for MRI (0.86–a0.89)^27^.

So far, spectral data for cartilage have mostly been acquired from animal cartilage^23–26^ or post-surgery samples of humans^28–31^, analyzing the infrared spectrum. The changes in the absorption of several wavelengths in this spectrum were found to correlate with degenerative changes in collagen and other components of cartilage. The authors proposed that the amount of water in the cartilage would be a valuable parameter for the degree of cartilage degeneration. However, they were not able to analyze the tissue's water content, as the samples used were ex vivo and already dehydrated^23,32^. Hence, some authors see a great potential in in vivo spectral analysis of cartilage to detect degenerative changes early^23^, and suggested that the NIR-spectrum would be useful^33^. It was found that NIR-spectroscopy was a valuable method to distinguish low-grade lesions from healthy cartilage^34^. Our findings are consistent with the previous studies, as the TWI value showed significant intraindividual differences between healthy and damaged cartilage. MRI also uses differences in water content to differentiate that^5^. However, the interindividual spread was too large to find a pragmatic TWI threshold value for clinical practice.

We found the relative absorbance at 540 nm to be a relevant parameter for the evaluation of cartilage tissue, using a threshold of > 0.74 to detect cartilage lesions of ICRS grade ≥ 3. This wavelength is within a spectral region that shows peaks for oxygenated hemoglobin^22^. We suggest that in higher grade cartilage defects, HSI might detect a peak in that spectral region, because the cartilage gets thinner, allowing the bone tissue underneath to influence the spectral data.

HSI is a very recent technique and, thus, still limited in its clinical applications. One limitation is, that HSI can only be used on the tissue surface, as its penetration depth is a few millimetres only^12^. For this study, the camera was used in open surgical approaches to the knee joint. For future applications, HSI needs to be combined and tested with arthroscopy or endoscopy to have a broader and more clinically relevant field of application. The results of this study are based on a sample size of 21 patients only. In order to confirm the preliminary findings of our data, studies with larger sample sizes are needed. This would allow for a more detailed differentiation of cartilage degeneration beyond the two extreme poles “healthy” and “damaged” and possibly more sophisticated information on the different “shades of gray” by the colourful spectra of Hyperspectral Imaging.

The ICRS grading system was used as the gold standard for determining the test accuracy of HSI. While the ICRS grading system is a well-validated standard, it is still just a tool to grade superficially visible defects of the cartilage. Future studies may add histopathology as a reference and the “gold standard” to be compared with the results of HSI.

In this cohort, 19 out of 21 patients were female and only cartilage of patients with knee surgery was included. This limits the external validity of our findings and different thresholds may apply for cartilage of the hip or shoulder, where the cartilage thickness is different. However, women are more often affected by cartilage degeneration than men and the most frequent localization of cartilage damage is the knee joint^35^. Future studies with a larger sample size that include different joints may confirm the results of this pilot study.

In summary, our study clearly shows that HSI has a high potential for the evaluation of cartilage tissue. Compared to MRI, it has the advantage of being available intraoperatively with only seconds needed for the measurements. Thus, the surgical procedure is only slightly disturbed and prolonged. It is completely contactless and radiation-free. No external contrast-medium has to be applied to the patient–aresulting in a highly effective and safe intraoperative diagnostic tool. Hopefully, it will be available through arthroscopy and endoscopy systems soon, as previously described by the LYSiS-system^36^. This would allow immediate intraoperative decision support for the surgeon when assessing a cartilage lesion and potentially change the operative procedure by this innovative “real time” modality.

## Conclusion

HSI can provide reliable parameters to differentiate between healthy and damaged cartilage. Therefore, it has the potential to serve as a valuable addition to arthroscopy, helping surgeons to identify critical lesions that are not visible with the naked eye.

Our data from a cohort of mainly elderly women suggest that the difference in absorbance at 540 nm is the best parameter to achieve accurate identification of damaged cartilage.

## Data Availability

All raw data can be provided by the corresponding author upon request.
